# The Role of Inositol in Thyroid Physiology and in Subclinical Hypothyroidism Management

**DOI:** 10.3389/fendo.2021.662582

**Published:** 2021-05-10

**Authors:** Salvatore Benvenga, Maurizio Nordio, Antonio Simone Laganà, Vittorio Unfer

**Affiliations:** ^1^ Department of Clinical and Experimental Medicine, Section of Endocrinology, University of Messina, Messina, Italy; ^2^ The Experts Group on Inositol in Basic and Clinical Research (EGOI), Rome, Italy; ^3^ Department of Experimental Medicine, “Sapienza” Università di Roma, Rome, Italy; ^4^ Department of Obstetrics and Gynecology, “Filippo Del Ponte” Hospital, University of Insubria, Varese, Italy; ^5^ Systems Biology Group Lab, “Sapienza” Università di Roma, Rome, Italy

**Keywords:** myo-inositol, subclinical hypothyroidism, thyroid dysfunctions, TSH, thyroid hormones

## Abstract

Myo-Inositol (MYO) is the most abundant stereoisomer of inositols’ family, cyclic polyols with 6 hydroxyl groups. Myo-Inositol has a relevant role in thyroid function and autoimmune diseases, as a precursor of phosphoinositides that takes part in the phosphatidylinositol (PI) signal transduction pathway. Among phosphoinositides, phosphatidylinositol 4,5- bisphosphate (PIP2) is the precursor of inositol triphosphates (IP3), second messenger of several hormones including thyroid-stimulating hormone (TSH). As a second messenger in the phospholipase C (PLC)-dependent inositol phosphate Ca^2+^/DAG pathway, Myo-Inositol is essential to produce H_2_O_2_ required for the synthesis of thyroid hormones. Consequently, depletion of Myo-Inositol or impaired inositol dependent TSH signaling pathway may predispose to the development of some thyroid diseases, such as hypothyroidism. Many clinical studies have shown that after treatment with Myo-Inositol plus Selenium (MYO+Se), TSH levels significantly decreased in patients with subclinical hypothyroidism with or without autoimmune thyroiditis. The TSH reduction was accompanied by a decline of antithyroid autoantibodies. Moreover, Myo-Inositol supplementation seemed to be involved also in the management of thyroidal benign nodules, with a possible effect in the size reduction. This review proposes a summary of the role of inositol, especially of Myo-Inositol, in the thyroidal physiology and its contribution on the management of some thyroid diseases.

## Introduction

The thyroid gland is responsible for the synthesis and the secretion of thyroid hormones (TH), triiodothyronine (T3) and thyroxine (T4), which is made up by the thyrocytes (epithelial cells that are also named thyroid follicular cells). Of the two hormones, T4 constitutes approximately 90% of the entire TH pool, while T3 makes up the remainder 10%. A peripheral conversion of the prohormone T4 into the biologically more active hormone T3 is made up by enzymes known as deiodinases, which produce around 80% of the total T3 (1). The deiodinases are homodimeric selenoproteins containing a thioredoxin fold, and classified in three types: type 1 (D1) and type 2 (D2) activate T4 into T3; type 3 (D3) inactivates both hormones (2). Specifically, among the activating enzymes, D2 has better catalytic efficiency than D1. TH circulate in the plasma bound mainly to three proteins: thyroxine-binding globulin (TBG), transthyretin (TTR) and albumin (3). Approximately 5% of TH circulate bound to lipoproteins (HDL> LDL> VLDL). Only approximately 0.03% of circulating T4 and approximately 0.3% of circulating T3 is free, nonprotein-bound (4).

After cell uptake by TH-binding sites on the plasma membrane, TH reach the nucleus and bind to specific receptors (TR) on the target cells, stimulating or inhibiting gene transcription (5). TH interact with all the biological systems, playing a key role for example in neurological development, in energy metabolism, and in cardiometabolic and reproductive systems.

Myo-inositol (MYO), which is an isoform of inositol, has been shown to play an important role in thyroid physiology. Clinical evidence indicated that patients with impaired thyroid functionality exhibit a higher demand of MYO than healthy subjects (6). MYO altered levels or impaired inositol dependent TSH signaling pathway, may predispose to the development of some alterations in thyroid functionality, such as hypothyroidism, pointing out MYO crucial role in preserving thyroid physiology, in increasing iodine availability and in counteracting its dysfunctions (7).

The aim is to review the role of MYO in the physiology of thyroid function and its potential use in the management of subclinical hypothyroidism (SCH).

## Mechanisms and Key Factors Involved in the Synthesis of Thyroid Hormones

TH biosynthesis occurs at the interface between the follicular lumen and the apical plasma membrane of thyrocytes, and it depends on the interaction of essential components: iodine (I_2_), a H_2_O_2_-dependent peroxidase called thyroperoxidase (TPO), and thyroglobulin (Tg), that works as iodine acceptor.

Iodine homeostasis in thyroid is guaranteed by iodine intake through the diet or food supplements, with a suggested daily intake of 150 µg for non-pregnant adults (8), providing values 20 to 50 times higher than the 10 µg/L in the plasma (9). After intestinal adsorption, iodide enters the thyroid through the sodium/iodide symporter (NIS), a transport protein located in the basolateral plasma membrane of thyrocytes, and then it enters in the follicular lumen crossing the apical membrane through a carrier called Pendrin, which shared with NIS a local amino acid sequence homology. Once in the thyroid follicular lumen, iodide is incorporated into the Tg, which is the predominant protein in the thyroid. Human Tg is a high molecular weight (660 kDa) glycoprotein homodimer (330 kDa each dimer) containing 66 tyrosine residues, but only up to one-fifth of them are iodinated, depending on the availability of dietary iodine.

Another crucial component in TH biosynthesis is the TPO, which is included in transport vesicles, which then merge with the apical plasma membrane of thyrocytes exposing the TPO catalytic site, with the attached heme group, in the thyroid follicular lumen (10). TPO incorporates iodine into Tg using H_2_O_2_ as final electron acceptor. Previous studies underlined that the enzyme responsible for the H_2_O_2_ synthesis is a membrane-bound NADPH-dependent oxidase (NOX), which exploits H_2_O_2_ as electron acceptor (11). The main system responsible for the H_2_O_2_ generation is composed of dual oxidases 1 and 2 (DUOX1 and 2) that are regulated by two different pathways: DUOX1 is activated by the protein kinase A through the G_s_PKA pathway; DUOX2 is regulated by protein kinase C through the G_q_-phospholipase C (PLC) pathway (12). To mediate the correct production of H_2_O_2_ the DUOX systems require maturation factors, called DUOXA, identified by *in silico* screening of multiple parallel signature sequencing data bases (13). Defects in DUOX and/or DUOXA represent possible causes of congenital hypothyroidism (CH), confirming the crucial role of the system (14). Besides its central role in TH biosynthesis, H_2_O_2_ is a toxic metabolite, therefore several defense mechanisms contribute in counteracting and avoiding oxidative stress related damages in thyroid (15).

Once all the elements are present at the interface of the follicular lumen and at the apical plasma membrane of thyrocytes, the hormonogenesis process can occur in the follicular lumen.

More specifically, TH synthesis comprises the following steps: 1) oxidation of TPO by H_2_O_2_; 2) oxidation of iodide ions by the TPO; 3) iodination of tyrosyl residues on Tg to form iodotyrosine moieties; 4) oxidation and coupling of iodotyrosine residues to form the final hormones, T3 and T4.

**Figure 1:**
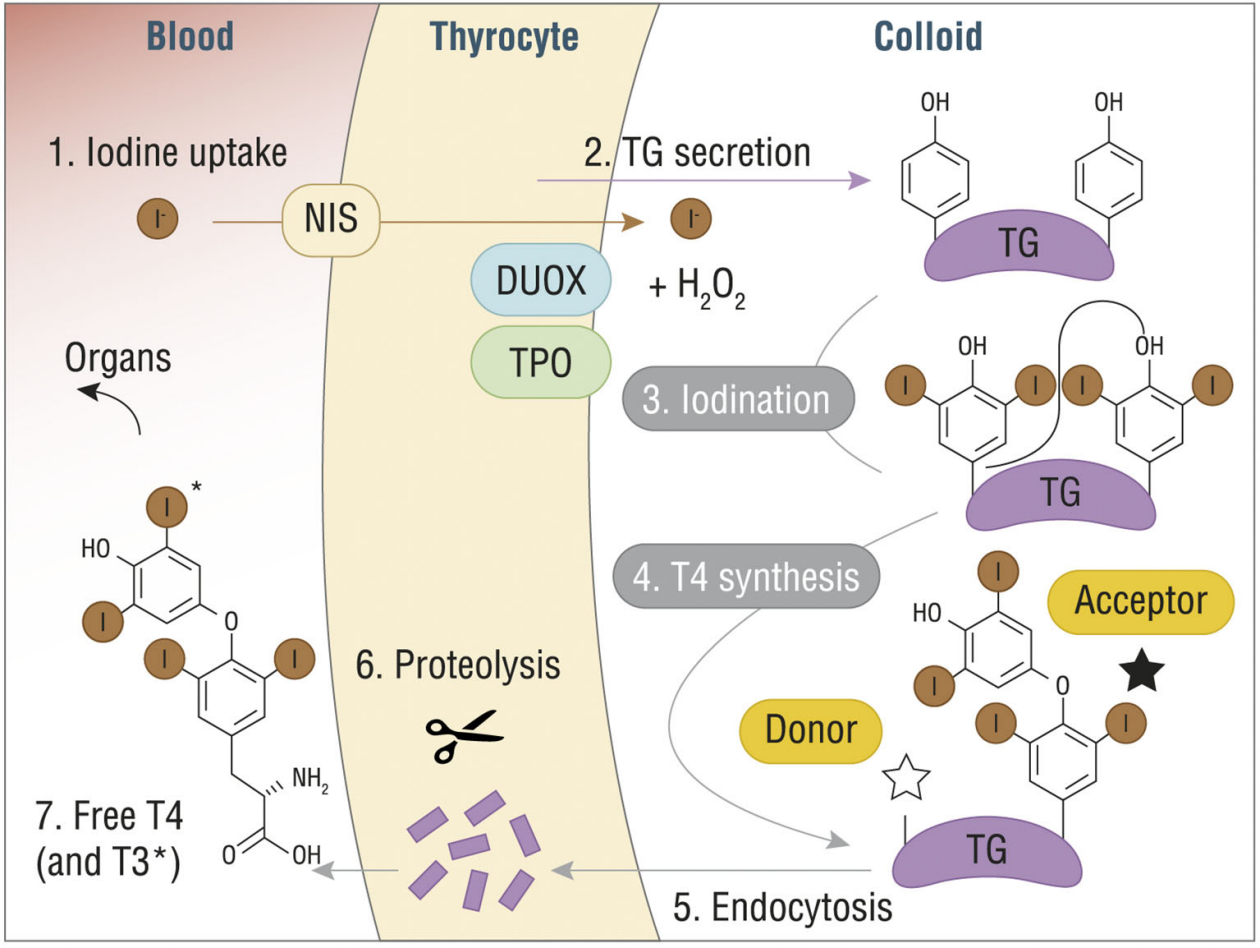
T4 (or T3) synthesis from Tg in thyroid gland. DUOX, dual oxidase; H_2_O_2_, hydrogen peroxide; I^-^, iodine; NIS, sodium/iodide symporter; T3, triiodothyronine; T4, thyroxine; TG, thyroglobulin. Reproduced with the permission from “The structure of human thyroglobulin.” Coscia F. et al., Nature 578.7796 (2020): 627-630 (License No 4998080013478).

After the incorporation of oxidized iodide into the Tg, through a process of iodination or iodine organification that generate either monoiodotyrosine (MIT) or diiodotyrosine (DIT) residues, the final step consists in coupling neighboring residues to form the final hormones. Specifically, coupling of two DIT residues yields T4, while coupling of DIT with MIT yields T3. TPO and H_2_O_2_ catalyze the coupling reaction, which is strictly dependent on Tg structure and leads to Tg-bound THs (**Figure 1**). A vescicle-mediated endocytosis, also known as micropinocytosis, or macropinocytosis, depending on the physiological circumstances, transports the modified Tg inside the thyrocytes, where MIT, DIT, and TH are released after proteolysis, crossing the basolateral membrane (16).

## The Role of TSH in the Control of Thyroid Hormone Synthesis

Iodide availably and thyrotropin (or thyroid stimulating hormone, TSH) are the main control mechanisms of TH synthesis. Low iodide availability may lead to inadequate TH synthesis, while excess iodide may completely inhibit their production. Such protective process, known as Wolff-Chaikoff effect, seems to inhibit H_2_O_2_ production and consequently Tg iodination.

TSH controls various steps of the synthesis and release of TH, starting from stimulating the NIS-mediated iodide uptake by the thyrocytes. TSH is synthesized by the anterior pituitary, under the stimulus of the thyrotropin-releasing hormone (TRH), which is secreted by the hypothalamus in a negative feedback system (17).

**Figure 2:**
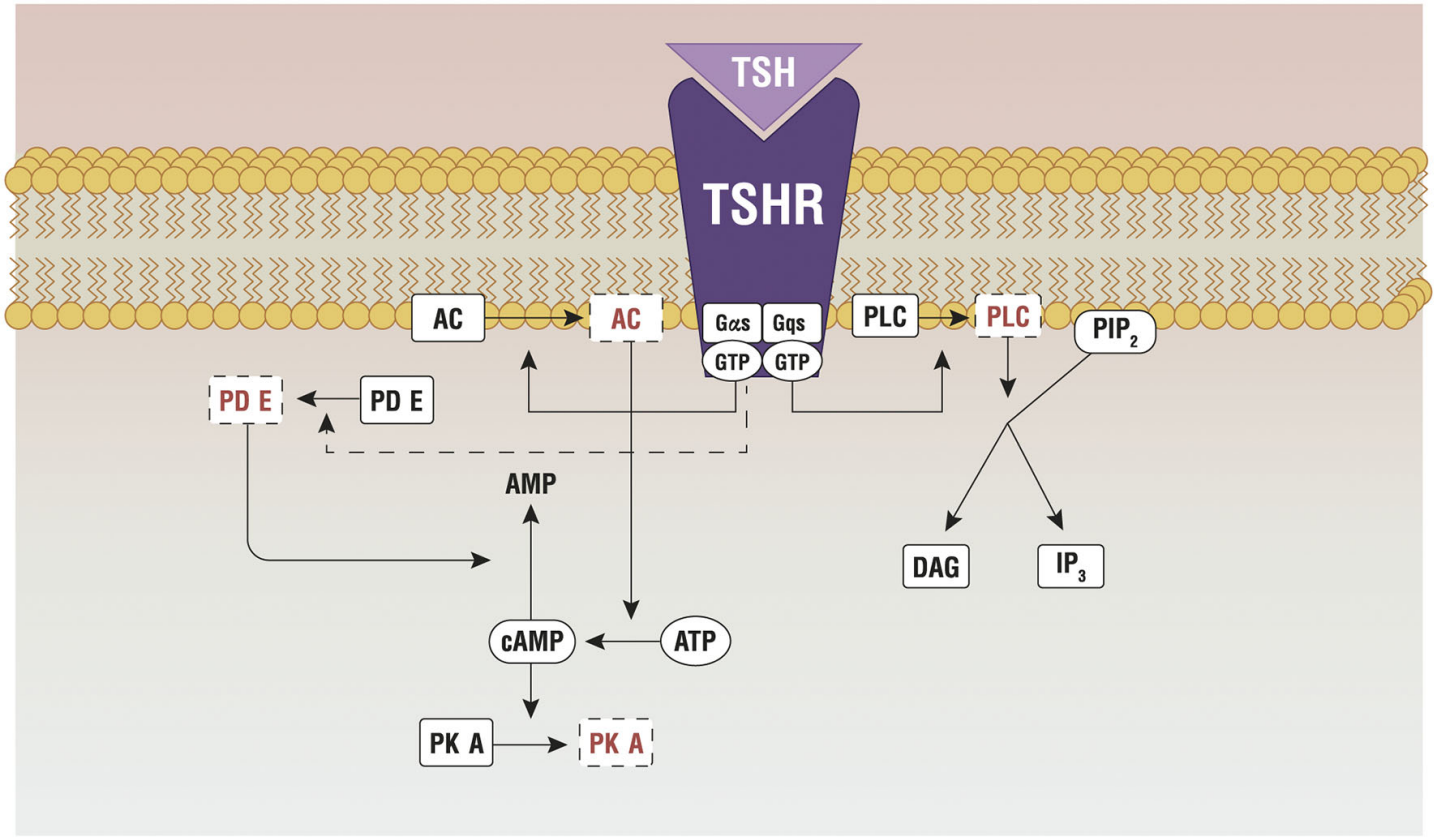
Principal pathways active by the TSH-TSHR binding. AC, adenylate cyclase; AKT, protein kinase B; ATP, adenosine triphosphate; cAMP, cyclic adenosine monophosphate; Gαs/Gqs, G protein; GTP, guanosine triphosphate; IP3, inositol triphosphate; mTOR, protein kinase (mammalian target of rapamicin); PDE, phosphoesterase; PIP2, phosphatidylinositol 4,5- biphosphate; PKA, protein kinase A; PLC, phospholipase C; TSHR, TSH Receptor; TSH, thyroid stimulating hormone. Reproduced with the permission from Benvenga et al. (23) (License No 4997560949993).

TSH stimulates TH synthesis by binding to its receptor (TSHR), which is expressed in the basolateral membrane of thyrocytes. TSHR is a G protein-coupled receptor (class A) that belongs to the sub-family of the glycoprotein hormone receptors (GPHRs), along with the gonadotropin receptors: the follicle-stimulating hormone receptor (FSHR) and the luteinizing hormone/choriogonadotropin receptor (LHCGR). Upon TSH binding, TSHR activates different G protein subtypes and signaling pathways, among which G_s_- and G_q_-induced signaling are probably the most important (18). G_sα_ signaling involves the activation of the adenylate cyclase, with production of cyclic adenosine monophosphate that phosphorylates protein kinase A (PKA). The pathway controls the expression of NIS, Tg, TPO and TSHR, through thyroid-specific transcription factors (19), and regulates Tg macrocytosis and internalization (20). G_q/11_ signaling encompasses the activation of PLC-dependent inositol phosphate Ca^2+^/DAG pathway, which leads to increased iodination through the release of inositol triphosphate (IP3) (21, 22). IP3, indeed, is responsible for the Ca^2+^ release from the endoplasmic reticulum, which is necessary for the activation of DUOX/DUAOXA2 system and the synthesis of H_2_O_2_ (**Figure 2**).

The two pathways are activated in separate moments, as they require different TSH concentrations. The cAMP signaling is 100 times more efficient to transmit the TSH signal than the inositol pathway. This is confirmed by an *in vitro* experiment on kidney embryonal cells expressing THSR (HEK-TSHR cells), where the authors demonstrate that the EC_50_ required to activate the cAMP signaling is 0.75 mU/mL, while EC_50_ ≥ 71 mU/mL is necessary for the inositol pathway (24). The presence of two binding sites on TSHR, with different affinity for TSH, explains such different behavior. The high-affinity site binds to TSH molecules at lower concentrations, activating the cAMP pathway; the low-affinity site binds to TSH at much higher concentrations and activates the inositol pathway (25).

## Subclinical Hypothyroidism

TSH is the leading, and sometimes the sole, diagnostic parameter that physicians use to evaluate the thyroid function.

Hypothyroidism is a condition where the thyroid functionality is lower than normal, and exists in two forms, overt and subclinical (SCH). The former is characterized by levels of TSH above the upper normal limit and low concentrations of THs, indicating thyroid insufficiency. On the other hand, SCH features TSH levels above the upper normal limit, but levels of TH within the normal range. This normality of TH levels occurs because small fluctuations of free levothyroxine (fT4) levels, although in a range that is normal for the population sampled to construe a reference range (but that it is less wide for any given individual), trigger the hypothalamic-pituitary-thyroid axis so as to increase the secretion of TSH (26). Scientists revised the upper physiological TSH limit multiple times over the years; for instance, it is 4,5 mIU/mL according to the National Health and Nutrition Examination Survey (NHANES III) (27, 28). SCH with TSH above this threshold can be divided in two types: *grade 1*, with TSH < 9.9 mIU/mL TSH; and *grade 2*, with TSH ≥ 10 mUI/mL (28).

SCH is a common condition especially in women, with a prevalence higher than 20% in subjects over 75 years (29). Autoimmune thyroid diseases (AITDs) are the main cause of SCH, with Hashimoto thyroiditis (HT) being the most common. In fact, HT occurs in approximately 5% of the Caucasian population and it is associated to 10-15% of SCH cases (30). Furthermore, SCH is at risk of progression to the overt form.

Also, long term effects of SCH are numerous and may involve many organic systems, with cardiometabolic, neurological, renal and reproductive issues. Indeed, SCH is associated with increased relative risk (RR) of cardiovascular disease (CVD) of around 1.33 (95% CI, 1.14-1.54). RR for the all-cause mortality is equally increased in patients with SCH (RR: 1.20; 95% CI, 1.07-1.34) (31). Hypothyroidism is also the second cause of dyslipidemia, characterized by elevated levels of low-density lipoprotein (LDL), cholesterol and triglyceride (32). SCH patients might also exhibit a major exposure to the development of nonalcoholic fatty liver disease (NALFD) (33), and reproductive issues. Female teenagers with thyroid problems are 4 times more likely to develop menstrual cycle disorders than their healthy peers. In particular, menstrual disturbances affect around 10.2% of SCH patients (34). A high TSH levels may also alter the ovarian reserve, thus reducing fertility (35). Moreover, elevated TSH seems to correlate with negative outcomes in pregnancy, increasing the rate of miscarriages and preterm births (36, 37).

Despite these long-term risks, the management of SCH still remains highly debated. The standard pharmacological treatment for hypothyroidism is based on levothyroxine (synthetic T4), due to its efficacy, long-term benefits, and good adsorption (38). However, some patient subgroups referred a slight preference for the combined therapy based on L-thyroxine and L-triiodothyronine (T3) (39), opening still unresolved issues in the management of hypothyroidism. Indeed, common variations in the gene codifying for the deiodinase type 2 may be responsible for both poorer psychological well-being on T4 monotherapy and improved response to combined T4/T3 therapy in patients on thyroid hormone replacement (40). On the other hand, studies comparing monotherapy with combined therapy, pointed out that subjects were unable to distinguish treatment and although as many as 18% preferred the combined approach, there was a relatively high incidence of side effects among those using the combination, making the topic more controversial (41, 42). In addition, as reported in the international guidelines drawn up by the American Thyroid Association Task Force on Thyroid Hormone Replacement, there is no consistently strong evidence of greater efficacy of the combined therapy over monotherapy with levothyroxine (43), confirming the monotherapy as the mainstay of treating hypothyroidism.

Noteworthy, current practice guidelines, which however produced many controversies, restrict the use of levothyroxine in the SCH to patients with overt hypothyroidism and TSH >10 mIU/mL. Indeed, one of the most critical problems related to SCH management, is the over treatment even in those patients for which it is not necessary. Pharmacological treatment of SCH is recommended only in particular cases, such as women seeking pregnancy and patients with important comorbidities (38, 43, 44), and it should be tailored to the individual patient based on the exposure at specific risk factors.

## Myo-Inositol

Inositol, a cyclic polyol with 6 hydroxyl groups, extracted, was isolated for the first time from muscle tissue by Schererer in 1980 (45). Inositols may exist in 9 possible isoforms (46). Myo-inositol (MYO) was the first isoform identified and it is the most abundant (more than 99%) in the eukaryotic cells (47). Dietary intake is the main source of MYO, either as free form or as phytate (IP6) (48). IP6 is more common in vegetables, while free inositol is more commons in animal sources. Dietary IP6 is metabolized by bacterial phytases (homologous of the mammalian InsP6 phosphatase) to produce free MYO, orthophosphate, and inositol-phosphate derivatives (i.e. mono-, di-, tri-, tetra-, and penta-phosphate esters) (49, 50). Fresh fruits, vegetables, beans and cereals are valuable inositol sources. In particular, large amounts of phytates are present in dried nuts, such as almonds, walnuts, and Brazilian nuts (9.4, 6.7 and 6.3% of dry weight, respectively) (49).

Dietary MYO and derivates are absorbed in the gut through sodium-dependent transporters, called sodium/myo-inositol channels type 1 (SMIT1) and type 2 (SMIT2), which are located in the duodenum and jejunum (51).

The Western diet guarantees a daily intake of around 1 g of MYO, but the absorption may be affected by a number of factors such as age, use of medications or substances like caffeine (52, 53).

Besides dietary intake, MYO is produced endogenously from glucose in two enzymatic steps. A hexokinase transforms glucose into glucose-6-phosphate, which is then converted into myo-inositol-1-phosphate. In humans, this endogenous synthesis produces up to 2 g MYO per day in each kidney, for a daily total of 4 g (54). Briefly, MYO homeostasis depends on three distinct mechanisms: 1) intestinal absorption and urinary excretion; 2) transport through specific carriers from plasma to interstitial fluid of cells; 3) endogenous synthesis and catabolism.

Exogenous administration of MYO in daily dosages of 4-30 g up to 12 months is generally well tolerated. Mild side effects, such as nausea and diarrhea, may appear only for daily dosages greater than 12 g (55).

**Figure 3:**
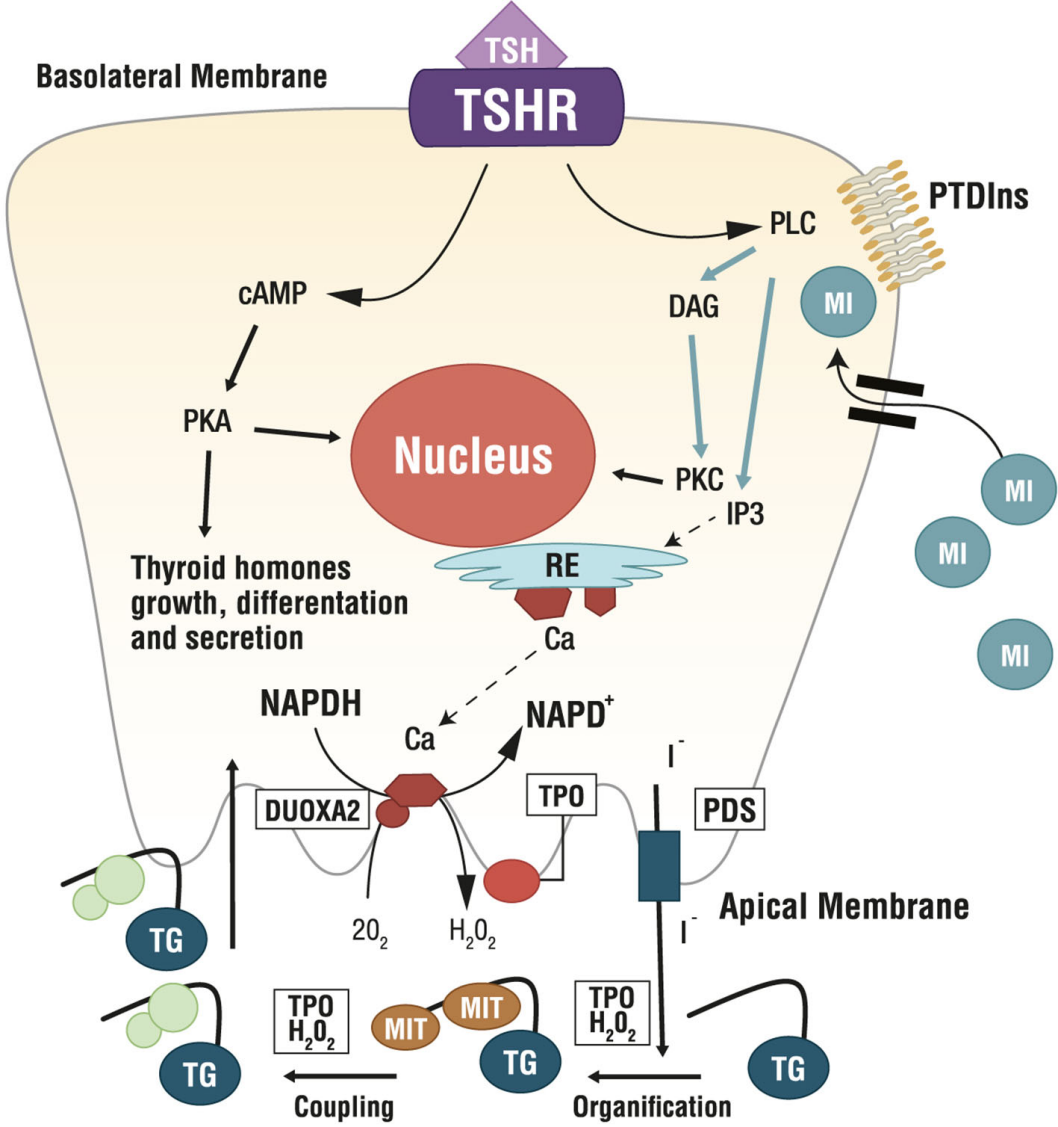
Role of Myo-Inositol in physiology of thyroid. Ca, calcium; cAMP, cyclic adenosine monophosphate; DAG, diacylglycerol; DUOXA2, dual oxidase A 2; ER, endoplasmic reticulum; H_2_O_2_, hydrogen peroxide; I^-^, iodine; IP3, inositol triphosphate; MI, myo-inositol; MIT, monoiodotyrosine; NAPD+/NADPH, nicotinamide adenine dinucleotide phosphate; O_2_, oxygen; PDS, pendrin; PKA, protein kinase A; PKC, protein kinase C; PLC, phospholipase C; PTDIns, phosphatidylinositol; TG, thyroglobulin; TSH, thyroid stimulating hormone; TSHR, TSH Receptor; TPO, thyreoperoxidase.

Several MYO-containing phospholipids are precursors for the biosynthesis of countless molecular intermediates involved in signaling transduction, including inositol triphosphate (IP3), inositol phosphates (IP), phosphatidylinositol (PI), phosphatidylinositol-phosphates (PIPs), glycosylphosphatidylinositols (GPIs), inositol-phosphoglycans (IPGs) and inositol ethers and esters (54, 56). Among these, phosphatidylinositol-4,5-biphosphate (PIP2) is the precursor of IP3 and diacylglycerol (DAG), which are involved in the phospholipase C (PLC)-dependent inositol phosphate Ca^2+^/DAG, working as second messengers of several hormones including TSH, LH, FSH and insulin (23) (**Figure 3**).

Therefore, it is not surprising that impaired MYO homeostasis correlate with a wide variety of conditions, including thyroid disorders, polycystic ovary syndrome (PCOS), fertility disorders, diabetes, metabolic, and neurological disorders (57).

Researchers found a correlation between intracellular MYO concentration and systemic TSH. Indeed, *in vitro* experiments demonstrated that thyrocytes actively accumulate MYO with increasing TSH levels (58). In thyrocytes, TSH dose-dependently stimulates inositol phosphate formation (59), with both the PIP2 cascade and the cAMP cascade controlling the synthesis of thyroid hormones. In the thyroid, imbalances in the inositol metabolism can impair thyroidal hormone biosynthesis, storage and secretion (23). Specifically, MYO regulates the H_2_O_2_-mediated iodination in thyrocytes through the PLC-dependent inositol phosphate Ca^2+^/DAG pathway, resulting in increased H_2_O_2_ generation, which is a crucial step for iodine organification and thyroid hormones biosynthesis. Indeed, H_2_O_2,_ generated under the stimulus of MYO, is available for iodine incorporation inside the thyroid (60, 61). Differently, the cAMP cascade induced by TSH activity, is more involved in cell growth and differentiation, and in thyroid hormones secretion. Therefore, MYO exerts a crucial role in thyroid physiology, by its function in TSH regulation of iodination and by an increased sensitivity of thyrocytes to TSH. Metabolomic investigations confirmed such evidence, indicating that MYO demand is higher in hypothyroid patients than in healthy subjects (6), making MYO very appealing as a molecule to increase iodine availability counteracting thyroid dysfunctions (7).

## Myo-Inositol in the Treatment of Subclinical Hypothyroidism

MYO is a second messenger in the phospholipase C (PLC)-dependent inositol phosphate Ca^2+^/DAG pathway, which leads to the production of H_2_O_2_ required for the synthesis of TH. Consequently, depletion of MYO or impaired inositol-dependent TSH signaling pathway may predispose to the development of hypothyroidism (62). In the last years, a growing interest for the role of MYO in thyroid pathophysiology has fostered new studies on its possible involvement in SCH and AITDs. In 2013, Nordio M. and Pajalich R. investigated the effectiveness of MYO oral treatment in women with AITD-related SCH. Forty-eight participants, with TSH comprised between 4.01 and 9.99 mIU/L and with positive TPOAb and TgAb, were randomly divided in two equal groups and treated either with 600 mg of MYO plus 83 μg of selenium (MYO+Se) or only 83 μg of selenium (Se) for 6 months. The authors found significant improvements of thyroid parameters in the MYO+Se group at the end of the study: 31% decrease of TSH (4.4 ± 0.9 vs 3.1 ± 0.6 mIU/mL), 44% decrease of TPOAb and 48% decrease of TgAb (p<0.01 for each parameter). In contrast, women in the Se group experienced improvement only in the antibody levels, with no change in the TSH level (63). Subsequent studies confirmed these findings. Morgante et al. investigated the prevalence of SCH in insulin resistant PCOS patients and the possible effect of inositol as insulin sensitizer. After 6 months of inositol + metformin treatment, TSH decreased significantly (4.00 ± 1.98 to 2.35 ± 1.65 mU/L), compared to metformin only (64).

More recent studies evaluated the effect of MYO treatment in a larger cohort of patients. A clinical trial on 86 patients with SCH and HT, who were treated with 600 mg MYO and 83 µg Se for 6 months, demonstrated a significant improvement in TSH levels (3.12 ± 0.09 mIU/L down from 4.32 ± 0.06 mIU/L at baseline; p ≤ 0.001). In addition, these patients reported a significant improvement in their quality of life, evaluated through a subjective symptomatology test (65). Another recent study included 168 patients with HT and TSH level between 3 and 6 mIU/mL. Participants were divided in two groups and treated either with MYO+Se (600 mg + 83 µg, respectively) or Se (83 µg). The authors found a significant improvement in thyroid parameters (TSH, fT4, TPOAb, TgAb) only in the MYO+Se group (66). Taken twice a day, this combined treatment was also effective in reducing TSH levels and the consequent risk to develop SCH (67). In 2018, another study demonstrated the efficacy and the safety of MYO+Se supplementation in pregnancy women. Patients with TSH comprised between 1.6-2.5 µIU/ml were enrolled and were treated with 600 mg MYO plus 83 µg Se, once a day, from the first to third trimester. The results showed a prevention against SCH thank to the treatment, with a stabilization of TSH, fT3 and fT4 (68).

Other studies assessed the efficacy of MYO in patients with SCH and HT, depending on the duration of the treatment, and found that MYO+Se supplementation reduced TSH by 21% in three months (69). TSH decreased further, and linearly, when treatment duration was extended up to 1 year (62).

Besides improving TSH levels (indicating improved TH-synthesis by the thyrocytes), MYO improves thyroid antibody levels, indicating ameliorations of the autoimmune process. HT is characterized by increased levels of Interferon γ (IFN-γ), which stimulates natural killer cells and lymphocytes CD4+ and CD8+ to secrete CXCL10 cytokine. CXCL10 is an important inflammatory marker for the thyroid, since it causes a strong inflammatory response that damages thyroidal morphology and functionality (70).

MYO+Se treatment demonstrated *in vitro* a protective effect on blood mononuclear cells (PBMC) from either HT or healthy patients, stressed with H_2_O_2_. In these experiments, MYO+Se decreased the expression of the cytokines CXCL10, CCL2 and CXCL9, with 80% reduction of the total cytokines. MYO+Se treatment dose-dependently improved cell vitality and Comet score, thus reducing genotoxicity (71–73).

Recent preliminary metabolomic investigations compared histological thyroid samples of healthy subjects with those of patients with non-malignant nodular diseases, follicular adenoma and thyroid carcinoma, focusing on the potential role of MYO in thyroid nodules. The authors concluded that MYO reduction correlates with increased thyroidal tissue malignancy. The study identified MYO and scyllo-inositol as possible markers for thyroid malignancy (74). A subsequent retrospective study examined the effects of 600 mg MYO supplementation for 6 months on benign nodules [class I and II according to the AACE/ACE/AME Guidelines that outline the risk of malignancy of thyroid lesions (75)] in patients with SCH and HT. The authors found significantly positive results regarding diameter reduction (16.72 ± 1.32 vs 12.44 ± 1.81), number of mixed nodules for patients (1.39 ± 0.16 vs 1.05 ± 0.15) and elasticity (1.80 ± 0.13 vs 1.24 ± 0.18) (76).

## Conclusion

Subclinical hypothyroidism (SCH) is a condition of impaired thyroid functionality, which is characterized by TSH levels above the upper physiological limit, but TH levels within the normal range. To date SCH management still remains a largely debated topic, even though it is a condition at high risk of progression to overt hypothyroidism, further exposing affected patients to many long-term effects. At the same time, the use of pharmacological levothyroxine-based therapy in SCH is recommended only when it is strictly necessary, since it risks binding patients to a chronic pharmacological approach throughout their life. Therefore, the management of the SCH needs a proper monitoring, tailored to the affected individual patient. In this way, MYO administration results being an intriguing approach. The importance of the physiological role of MYO in the wellbeing of the organism is well recognized. As a precursor of second messengers, such as IP3, MYO supports the proper function of several organs and tissues, including the thyroid (**Table 1**). Specifically, since its crucial role in TSH regulation of iodination in the process of TH biosynthesis, MYO physiological levels correlate with a euthyroid condition. Numerous publications indeed demonstrate the beneficial effects of MYO treatment against subclinical hypothyroidism and autoimmune thyroiditis both *in vitro* and *in vivo*, pointing out MYO intriguing application in recovering thyroid dysfunctions. Specifically, the up-to-date picture of clinical results here reported, reveal that the supplementation MYO is useful to manage subclinical hypothyroidism conditions. This approach can avoid the progression to the overt hypothyroidism and delay the occurrence of a chronic pharmacological therapy in these patients, extending the therapeutic use of MYO and shaping future clinical studies on SCH management.

**Table 1:** Effects of inositol on thyroid functionality.

INOSITOL IN THYROID	
**Physiology**	Precursor of Inositol triphosphates (IP3), second messenger of thyroid-stimulating hormone (TSH) Modulation the phospholipase C (PLC)-dependent inositol phosphate Ca^2+^/diacylglycerol (DAG) pathway Modulation the Ca^2+^ release from the endoplasmic reticulum, which is necessary for the activation of DUOX/DUAOXA2 system and the synthesis of H_2_O_2_
**Pathology**	Thyrocytes actively accumulate MYO with increasing TSH levels MYO demand is higher in hypothyroid patients than in healthy subjects
**Treatment**	Decline of TSH in subclinical hypothyroidism patients Decline TPOAb and TgAb in autoimmune thyroiditis Decline expression of cytokines CXCL10, CCL2 and CXCL9 Reduction of diameter and number of mixed thyroid nodules

## Author Contributions

All authors listed have made a substantial, direct, and intellectual contribution to the work, and approved it for publication.

## Conflict of Interest

VU is an employee at Lo.Li Pharma s.r.l., Rome (Italy).

The remaining authors declare that the research was conducted in the absence of any commercial or financial relationships that could be construed as a potential conflict of interest.
